# Pseudosarcomatous myofibroblastic proliferation of the appendix with an abdominal abscess due to diverticulum perforation: a case report

**DOI:** 10.1186/s40792-020-00901-1

**Published:** 2020-06-22

**Authors:** Tetsuya Tanaka, Takeshi Ueda, Takashi Yokoyama, Suzuka Harada, Kinta Hatakeyama, Atsushi Yoshimura

**Affiliations:** 1 Department of Surgery, Minami-Nara General Medical Center, 8-1 Oaza-Fukugami, Oyodo-cho, Yoshino-gun, Nara 638-8551 Japan; 2 Department of Diagnostic Pathology, Minami-Nara General Medical Center, 8-1 Oaza-Fukugami, Oyodo-cho, Yoshino-gun, Nara 638-8551 Japan

**Keywords:** Pseudosarcomatous myofibroblastic proliferation, Appendix, Inflammatory pseudotumor

## Abstract

**Background:**

Pseudosarcomatous myofibroblastic proliferation is a rare proliferative lesion of the submucosal stroma characterized by myofibroblast proliferation and inflammatory cell infiltration, and is mainly reported in the urinary system.

**Case presentation:**

We report a 65-year-old male who was referred to our emergency room with right-side iliac fossa pain. The pain gradually worsened for approximately 2 months, and rebound tenderness was positive. Blood examination showed severe inflammatory findings, and enhanced computed tomography revealed a heterogeneous contrast-enhancing mass lesion measured to be 55 × 50 mm in size at the lower right abdomen. Based on these results, the patient was diagnosed with appendicitis with an abdominal abscess. As the inflammation was severe, we drained the abscess before performing surgery. Approximately 1 month after the abscess diminished, interval appendectomy was performed. Macroscopic findings of the resected specimen showed a perforated diverticulum of the appendix and a small adjacent nodule measured to be 14 mm in size. Histopathological examination with hematoxylin and eosin staining revealed that the nodule consisted of fibroblast proliferation and inflammatory cell infiltration. Furthermore, immunohistochemical examination showed positive for smooth muscle actin and desmin and negative for S-100, c-kit, and anaplastic lymphoma kinase. Based on these histopathological results, we diagnosed the nodule as an unusual case of a pseudosarcomatous myofibroblastic proliferation associated with perforation of the diverticulum of the appendix.

**Conclusion:**

Herein, we report a rare case of a pseudosarcomatous myofibroblastic proliferation that occurred in the appendix with diverticulitis.

## Background

Pseudosarcomatous myofibroblastic proliferation (PMP) is a rare disease entity of unknown etiology and pathogenesis. PMP is also known as an inflammatory pseudotumor (IPT) or inflammatory myofibroblastic tumor (IMT), and it is characterized by myofibroblast proliferation and inflammatory cell infiltration. It was first reported by Roth et al. in 1980 [1]. A common site for a PMP is the urogenital tract. Other sites where PMPs may occur include the head, neck, pelvic cavity, retroperitoneum, and abdomen [2]. The only report of a PMP in the digestive system is in the gallbladder [3], and there are no reports of a PMP in the gastrointestinal tract. This tumor has no features on imaging, blood test, or clinical findings, and it is difficult to diagnose preoperatively. According to the latest World Health Organization (WHO) classification [4], a PMP is a benign tumor with malignant potential derived from fibroblasts and myofibroblasts.

Herein, we present a rare case of a PMP that occurred in the appendix of a 65-year-old male presenting with an abdominal abscess due to perforation of the appendix diverticulum.

## Case presentation

**Fig. 1:**
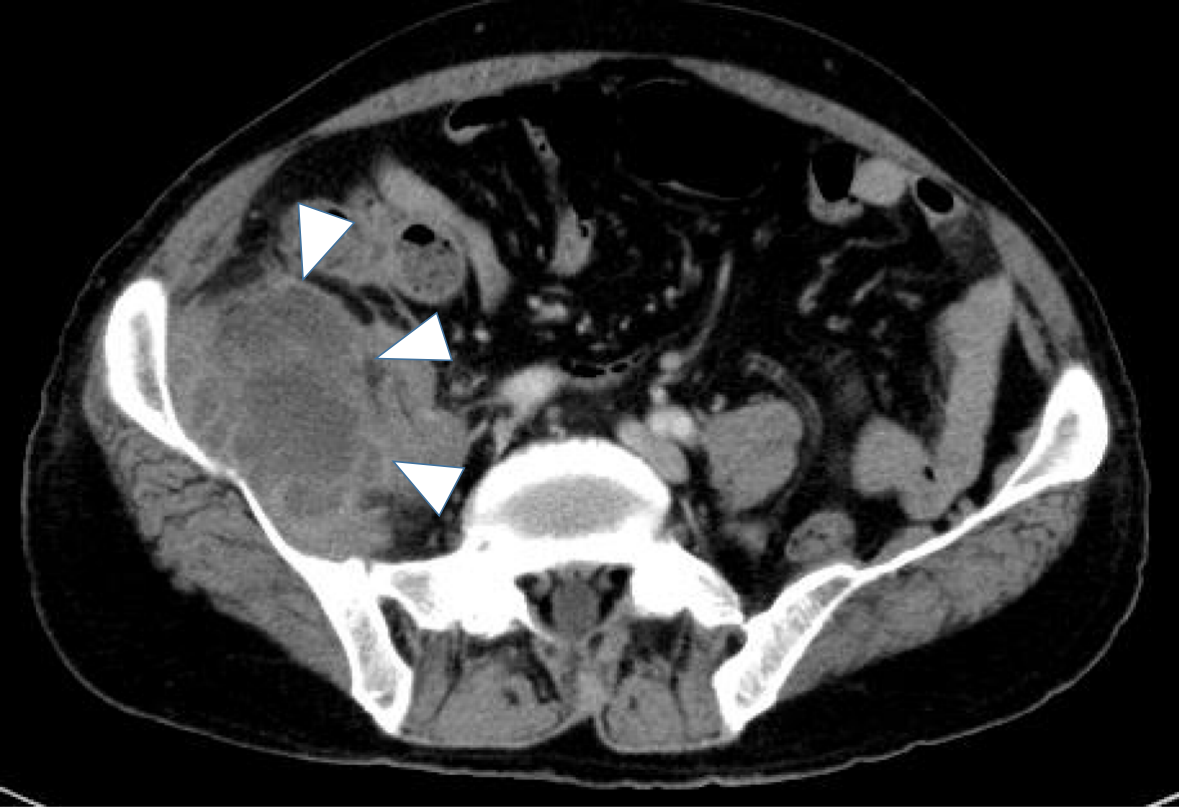
CT image. Enhanced computed tomography revealed a heterogeneous contrast-enhancing mass lesion measured to be 55 × 50 mm in size at the lower right abdomen (white arrow)

A 65-year-old man was referred to our emergency room with right-side iliac fossa pain. The pain had persisted for approximately 2 months and gradually worsened, and then he noticed a mass in the lower right abdomen that had increased in size. On physical examination, rebound tenderness was positive. The lump was approximately 50 × 40 mm in size, hard, and with tenderness on deep palpation. Blood examination showed severe inflammatory findings [white blood cell count, 16,500/μL (normal range 3590–9640/μL); C-reactive protein, 21.3 **mg/dL** (normal range 0.5–1.0 **mg/dL**)], but tumor markers such as carcinoembryonic antigen (CEA) and carbohydrate antigen 19-9 (CA19-9) were within the normal range. Enhanced computed tomography revealed a heterogeneous contrast-enhancing mass lesion measured to be 55 × 50 mm in size at the lower right abdomen (Fig. 1), and the border between the iliopsoas muscle and this mass lesion was unclear. Based on these results, the lesion was diagnosed as an abscess formation due to retroperitoneal perforation of acute appendicitis. Because the abscess had reached the iliopsoas muscle, we performed interval appendectomy [5] after abscess reduction with percutaneous drainage. Contrast examination using the percutaneous abscess drainage tube revealed continuity between the abscess cavity and the appendix. An appendectomy was performed after the abscess cavity had been significantly reduced and the inflammatory findings on blood tests had improved, approximately 1 month after the start of drainage.

**Fig. 2:**
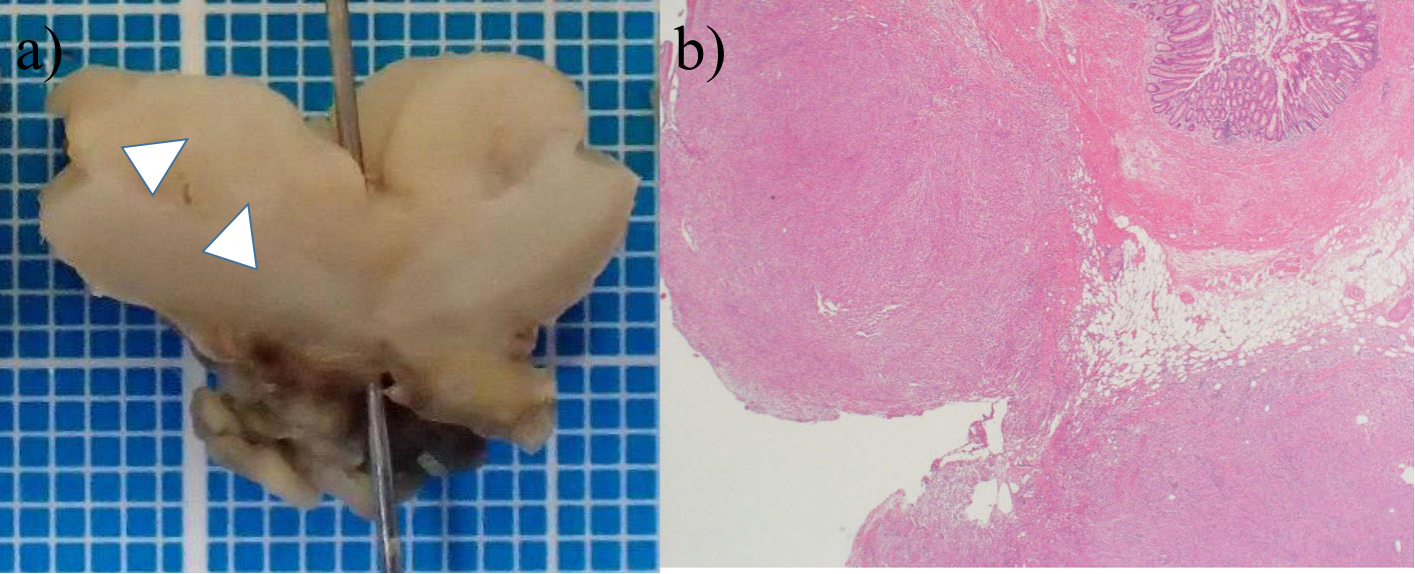
Findings of the resected specimen. **a** Macroscopic findings of the resected specimen showed a perforated appendiceal diverticulum (needle) and a small nodule measured to be 14 mm in size (white arrow). **b** Histological findings. Low-magnification view of the mass lesion

**Fig. 3:**
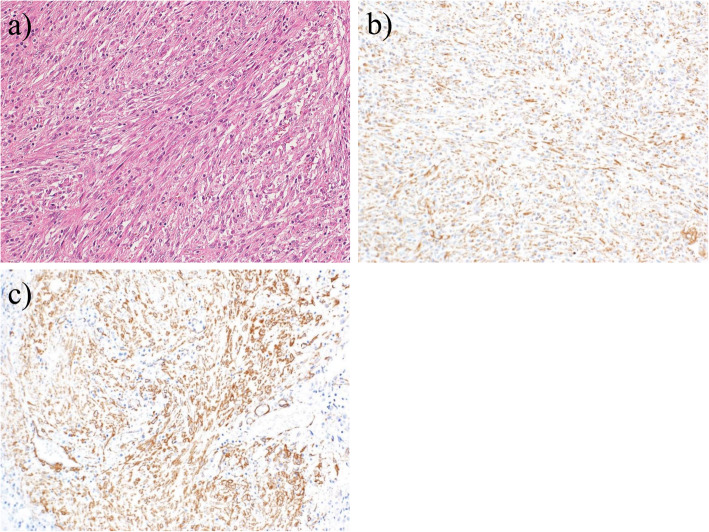
Histopathological findings. **a** Histopathological examination using hematoxylin and eosin staining revealed spindle cell proliferation and significant inflammatory cell infiltration, magnification × 200. **b**, **c** Immunohistochemistry showed that the spindle-shaped cells were strongly positive for vimentin (**b**) and smooth muscle actin (SMA) (**c**), magnification × 200

The resected appendix showed moderate swelling and inflammatory findings, as well as a diverticulum continuing to the abscess cavity. Macroscopic findings of the resected specimen showed a perforated appendix diverticulum and a small adjacent nodule measured to be 14 mm in size (Fig. 2a, b). Histopathological examination about the small nodule using hematoxylin and eosin staining revealed spindle cell proliferation and significant inflammatory cell infiltration composed mainly of lymphocytes and plasma cells (Fig. 3a). Moreover, immunohistochemistry was performed, and the spindle-shaped cells were strongly positive for vimentin (Fig. 3b) and CD68 (PGM-1) and focally positive for smooth muscle actin (SMA) (Fig. 3c). In contrast, S100, CD117 (c-kit), CD34, and anaplastic lymphoma kinase (ALK) were all negative. Based on these histopathological findings, the final diagnosis of this lesion was a PMP found in the appendix with appendiceal diverticulum perforation.

There were no postoperative complications such as abscess relapse, and the patient was discharged with good progress. This patient was followed up for 18 months after surgery, and he remained in good condition without any sign of local recurrence or distant metastasis.

## Discussion

Inflammatory pseudotumor (IPT) is a mass-forming lesion consisting of spindle cell proliferation and inflammatory cell infiltration, as reported by Umiker in 1954 [6]. PMP is a class of inflammatory pseudotumors first described by Roth in 1980. Besides IPT, many names have since been used to describe histologically similar lesions, including IMT, pseudosarcomatous myofibroblastic tumor, pseudosarcomatous fibromyxoid tumor, and myofibroblastic proliferation.

Among these, an IMT is similar to a PMP in histopathological findings and is characterized by myofibroblast proliferation and infiltration of inflammatory cells. Although a PMP is morphologically similar to an IMT, an IMT is mainly reported in the respiratory tract in children and young adults. Most reports of PMPs are in the urinary tract in young and middle-aged adults [7, 8], and the only report of a PMP in the digestive field is in the gallbladder [3]. To our knowledge, no previous cases of gastrointestinal PMP have been reported, and our case is the first to be reported in the gastrointestinal tract.

The diagnosis of PMP from clinical symptoms is difficult because PMP has no specific symptoms, regardless of the location. Furthermore, it is difficult to distinguish PMPs from other inflammatory diseases and mass lesions based on clinical symptoms, imaging tests such as computed tomography examination, and blood test findings. Therefore, the final diagnosis of PMP is dependent on the findings of postoperative histopathologic and immunohistochemical examinations. In our case, the preoperative diagnosis was an intraperitoneal abscess associated with appendicitis perforation. On pathological examination by staining with hematoxylin and eosin, the mass lesion was composed of spindle cell proliferation and inflammatory cells. In addition, immunohistochemistry revealed that the lesion was negative for S-100 and c-kit, which could rule out neurogenic tumors and gastrointestinal stromal tumors. Conversely, the lesion was positive for vimentin and SMA, suggesting that it was derived from fibroblasts or myofibroblasts. Clinically, differentiation from myogenic malignant tumors is important. In this case, malignant tumors such as leiomyosarcoma were excluded because the lesion did not have severe cytologic atypia and was less mitotic than sarcomas, and there was a lack of nuclear hyperchromatism and variation in shape and size of the spindle cells [9]. IMT is a similar disease to PMP and is characterized by myofibroblast proliferation and inflammatory cell infiltration. The importance of ALK expression in differentiating between IMT and PMP has been emphasized [10]. In our case, the infiltration of inflammatory cells was weaker than the infiltration of IMT and immunohistological findings were negative for ALK. In addition, we consulted a pathologist at another institution who specializes in soft tissue tumor pathology to evaluate the specimen. He responded that the pathological result of this nodular lesion should be reactive myofibroblastic proliferation. Therefore, we diagnosed the lesion as a PMP found in the appendix. However, the definitions of PMP and IMT remain controversial because their potential for malignancy is unclear [11]. Therefore, there are no clear guidelines for the differential diagnosis of inflammatory pseudotumors such as IMT and PMP. Currently, a diagnosis is made on a case-by-case basis.

It has been hypothesized that inflammatory pseudotumors such as IMT and PMP are produced as a result of inflammatory reactions such as surgery, trauma, infection, and tumors. Pettinato et al. published a large case series that included 42 cases of PMP, and they reported that some lesions developed spontaneously while others were secondary to trauma, surgery, or drugs [12]. It is possible that in our case, the PMP may have been caused by continuous chronic inflammation over a relatively long period of time due to perforation of the diverticulum of the appendix, which had spread to the retroperitoneum and formed an abscess.

Surgery is the main treatment strategy for IPTs such as PMPs and IMTs.

According to the WHO, an IMT has been defined as a low-grade malignancy tumor [4], and distant metastasis has been reported [2]. It is not yet clear whether a PMP is a genuine tumor, a reactive lesion, or even a proliferative mesenchymal lesion [13]. However, there are reports of local recurrence, even though a PMP is completely resected in many cases and the disease is considered to be a reactive inflammatory proliferation [13, 14]. The potential for malignancy is still unclear, and the issue is controversial [11]. Therefore, further research and postoperative surveillance are needed to establish a correct diagnosis of these lesions. Furthermore, it is necessary to clarify the clinical significance of this disease in the gastrointestinal tract by accumulating more cases and tracking the prognosis.

## Conclusion

To the best of our knowledge, no gastrointestinal reports of PMP lesions have previously been published. Thus, our case study may serve as a therapeutic experience if a similar case is encountered.

## Data Availability

The dataset supporting the conclusions of this article is available in the manuscript.

## Abbreviations

PMP: Pseudosarcomatous myofibroblastic proliferation
IPT: Inflammatory pseudotumor
IMT: Inflammatory myofibroblastic tumor
WHO: World Health Organization
CEA: Carcinoembryonic antigen
CA19-9: Carbohydrate antigen 19-9
CT: Computed tomography
SMA: Smooth muscle actin
ALK: Anaplastic lymphoma kinase
